# Staged Versus Concomitant Carotid Endarterectomy and Aortic Valve Replacement: A Case Report and Literature Review

**DOI:** 10.7759/cureus.49773

**Published:** 2023-12-01

**Authors:** Muhammad Faiq Umar, Shannay E Bellamy, Muhammad Ahmad, Muhammad Mirza, Ayesham Sitara, Michael Benz, Abdul A Ameen

**Affiliations:** 1 Internal Medicine, Mayo Hospital, Lahore, PAK; 2 Cardiology, Jersey City Medical Center, Jersey City, USA; 3 Internal Medicine, Jersey City Medical Center, Jersey City, USA; 4 Interventional Cardiology, Jersey City Medical Center, Jersey City, USA; 5 Cardiology, Rutgers New Jersey Medical School, Newark, USA; 6 Cardiology, Christ Hospital, Jersey City, USA

**Keywords:** coronary artery bypass grafting(cabg), combined cardiac surgery, tavr( transcatheter aortic valve replacement), carotid artery stenting (cas), cardiac valvular surgery, severe asymptomatic aortic stenosis, carotid endarterectomy (cea), aortic valve surgery, perioperative stroke, bilateral internal carotid artery stenosis

## Abstract

Stroke is a common complication of cardiac surgery, and carotid artery stenosis is an established risk factor for stroke. Therefore, patients with carotid artery stenosis who are undergoing cardiac surgery require proper management of the former either simultaneously or before cardiac surgery. We present a challenging case of a 67-year-old male patient who presented with generalized weakness, severe aortic stenosis, and significant bilateral carotid artery stenosis. The coexistence of these findings sparked a debate about whether to perform a carotid endarterectomy first or an aortic valve replacement. Moreover, a past history of percutaneous coronary intervention and coronary artery bypass grafts made the decision more challenging. Multiple approaches have been employed for the management of coexisting carotid artery stenosis with cardiac surgery; however, no definitive guidelines exist, especially for surgeries other than coronary artery bypass grafts or where the carotid stenosis is bilateral and severe.

## Introduction

Perioperative stroke, defined as a stroke occurring within 30 days following surgery, is a well-established complication of cardiac surgery [1]. Carotid artery stenosis (CAS) is prevalent in 6% to 12% of patients scheduled for cardiac surgery and increases the risk of perioperative stroke up to 9.2% [2-4]. Therefore, it should be managed simultaneously. However, multiple studies have reported conflicting evidence regarding the management strategy. The data is much more scarce, particularly for coexisting severe aortic valve stenosis and CAS; moreover, the emergence of novel therapies has made the appropriate choice of procedure more challenging. We came across this dilemma when a patient presented to our hospital with widespread peripheral arterial disease (PAD), advanced CAS, and severe aortic valve stenosis. We present this case and review existing literature to frame management strategies for patients with similar conditions.

## Case presentation

**Figure 1:**
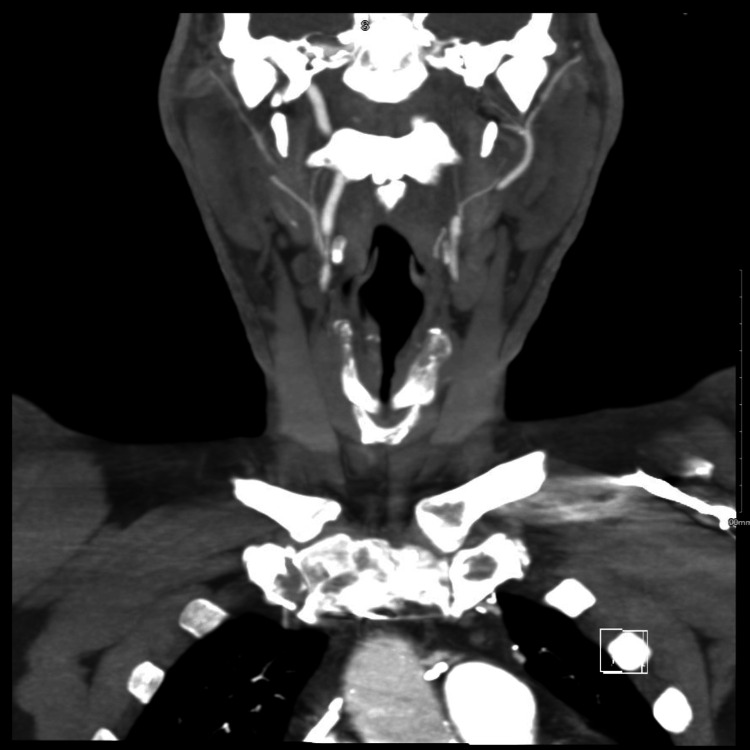
CT angiography illustrating severe bilateral carotid artery stenosis

A 67-year-old male with a smoking history of 52 pack years presented to our hospital in May 2023 to evaluate generalized body aches. His past medical history was significant for coronary artery disease, PAD, hypertension, hyperlipidemia, chronic obstructive pulmonary disease, and major depressive disorder. He had a history of multiple vascular procedures, along with percutaneous coronary intervention (PCI) with stenting and coronary artery bypass graft surgery (CABG). Except for an anterior midline scar, his physical examination was unremarkable. His laboratory reports were within the normal range (Table 1), while a chest X-ray did not reveal any active pulmonary disease. An electrocardiogram depicted the absence of p waves, irregularly spaced narrow QRS complexes, and a mean ventricular rate of 55 bpm, findings that are suggestive of atrial fibrillation with sick sinus syndrome. Noninvasive imaging studies revealed extensive PAD. The carotid duplex noted an occluded left internal carotid artery and 70% to 99% stenosis of the right internal carotid artery with antegrade flow in vertebral arteries (Figure 1).

**Table 1:** Laboratory results on admission MCV: Mean corpuscular volume, eGFR: Estimated glomerular filtration rate, AST: Aspartate aminotransferase, ALT: Alanine transaminase, INR: International normalised ratio

Laboratory test	Value	Reference range
Total leukocyte count (K/ul)	6.5	4-11
Erythrocyte count (M/ul)	4.89	4-5.9
Hemoglobin (g/dL)	12.4	14-18
Hematocrit (%)	41.4	40-50
MCV (fl)	84.7	80-100
Platelet count (K/ul)	203	130-400
Sodium (mmol/L)	139	136-145
Potassium (mmol/L)	4.5	3.5-5.1
Chloride (mmol/L)	108	98-107
Blood Urea Nitorgen (mg/dL)	10	9-23
Creatinine (mg/dL)	1.0	0.7-1.3
eGFR (ml/min)	82.5	>90
Glucose (mg/dL)	96	60-100
Calcium (mg/dL)	8.9	8.7-10.4
AST (units/L)	21	8-34
ALT (units/L)	15	10-49
Alkaline phosphatase (units/L)	54	46-116
Bilirubin (mg/dL)	0.4	0.3-1.2
Prothrombin time (sec)	12.7	12-15.1
INR	1.09	0.85-1.14
Partial thromboplastin time (sec)	36.1	25.4-36.7
Troponin I (ng/ml)	17.8	Within normal limits

The lower extremity arterial duplex illustrated >70% stenosis of bilateral common femoral arteries, 50% of the left superficial femoral artery, and occlusion of the right superficial femoral artery with distal reconstitution. Computed tomography angiography (CTA) confirmed these findings, along with bilateral external iliac artery occlusion. A transthoracic echocardiogram revealed low-flow, low-gradient aortic stenosis, an aortic valve area of 0.94 cm2, and a transaortic valve gradient of 29 mmHg.

A multidisciplinary conference was convened to develop a consensus on concomitant versus staged carotid endarterectomy (CEA) and aortic valve replacement (AVR). Given the lower risk of perioperative stroke and mortality with staged procedures, a decision was made in favor of prophylactic carotid endarterectomy followed by AVR. However, the patient left the hospital against medical advice because of the delays in definitive management.

## Discussion

Cardiac disease and carotid stenosis are often coexisting problems, originating from the same pathophysiology, but the management strategy has remained controversial over several years [4]. A wide spectrum of conflicting evidence has been published, so much so that Li et al. have reported that severe carotid stenosis does not increase the risk of postoperative stroke with cardiac surgery, hence eliminating the need for carotid endarterectomy with open heart surgery [5,6]. Contrarily, while some authors did report a higher incidence of perioperative stroke, they believe this was due to multifactorial etiology rather than the stenosis itself [7]. Similarly, while some authors believe that concomitant procedures involving CEA and CABG are a strong risk factor for perioperative stroke, others believe it is not the case [8,9]. The vast difference in these results can be attributed to multiple factors, including patient heterogeneity, selection criteria, non-randomization, retrospective design, and various biases.

While most authors support the need for carotid revascularization with open heart surgery, whether to do a staged or a concomitant endarterectomy remains controversial. Donmez et al. reported no significant difference between staged or concomitant procedures [10]. Although some authors reported the superiority of the staged procedure [11,12], others find a synchronous or concomitant procedure acceptable [13-17]. Even with the staged approach, whether to do a prophylactic CEA or a delayed one is debatable, with most studies supporting the superiority of CEA before cardiac surgery [18-21].

Tzoumas et al. performed a meta-analysis to evaluate staged versus synchronous CEA and CABG [22]. They reported that patients in the simultaneous group had a significantly higher risk of 30-day mortality and stroke but a lower risk for myocardial infarction as compared to the staged group. The rates of transient ischemic attacks, postoperative bleeding, and pulmonary complications were similar between the two groups. However, the studies included were only observational ones, often retrospective and non-randomized, and did not have stringent selection criteria. Moreover, these data are specifically for CABG and should not be generalized to all cardiac surgery procedures.

The CABACS trial published in 2022, revealed that patients with asymptomatic CAS undergoing combined CABG and CEA had a similar long-term risk of stroke or death as compared with CABG alone, but considering the high perioperative risk of the combined procedure, CABG alone should be preferred in patients with asymptomatic CAS; however, the sample size was small and it wasn't adequately powered to reach statistically significant results [23].

The choice of procedure becomes further perplexing with the advent of novel approaches. Carotid artery stenting can be used as an alternative to CEA. Naylor et al. documented a comparable 30-day risk of death and any stroke for staged carotid artery stenting, CABG, and CEA-CABG [24]. Moreover, Giannopoulos et al. reported significantly higher 30-day mortality in the CEA + CABG group as compared to carotid artery stenting + CABG [25]. Even within the carotid artery stenting group, whether to do a staged procedure or a synchronous one remains debatable. In another meta-analysis including four observational studies comparing staged and concomitant carotid artery stenting and CABG, Tzoumas et al. reported an increased risk of 30-day stroke in the concomitant group as compared to the staged one [26].

Our patient was also unique in the sense that he had bilateral severe carotid artery disease, i.e., an occluded left and severely stenosed right side. Bilateral carotid artery disease is an adverse prognostic factor for neurologic complications, increasing the risk of stroke three-fold and up to five-fold if occluded [27,28]. Lescan et al. reported that combined CEA and cardiac surgery in the presence of a carotid occlusion had a significantly higher stroke rate than isolated cardiac surgery [3]. Therefore, the authors recommended considering staged prophylactic CEA/CAS for patients with bilateral asymptomatic carotid artery stenosis [3,29].

Furthermore, most of the data in the literature exists specifically for CABG and CEA, but our patient had to undergo an AVR Limited data exists on patients undergoing isolated AVR and CEA. Yoda et al. reported that AVR could be safely performed along with CEA using cardiopulmonary bypass for both procedures [30]. They found age over 70, double valve surgery, and myocardial infarction as independent risk factors for mortality.

Moreover, our patient already had a previous history of open heart surgery (CABG). Gupta et al. reported that, compared with surgical AVR, transcatheter aortic valve replacement (TAVR) is associated with lower rates of in-hospital complications in patients with a prior history of CABG [31]. However, limited data exists for concomitant TAVR and carotid artery stenting, or CEA. Moraca et al. reported favorable outcomes for 16 patients who underwent concomitant CEA and TAVR [32]. Similarly, cases have been reported where trans-carotid artery revascularization was done along with TAVR [33] (Table 2). However, these sample sizes are small, and more data is required to frame definitive strategies. It is high time for an appropriately powered randomized controlled trial comparing these several management strategies in patients with varying degrees of stenosis and symptoms.

**Table 2:** Studies examining aortic valve replacement and carotid endarterectomy AVR: Aortic valve replacement, CEA: Carotid endarterectomy

Study	Procedure	Isolated AVR and concomitant CEA (n)	Mortality (n)	Stroke (n)
Yoda et al. (2004)^[30]^	Valve replacement + CEA	64	3	6
Gansera et al. (2012)^[27]^	Cardiac surgery + CEA	31	Not reported separately	Not reported separately
Snider et al. (2000)^[14]^	Cardiac surgery + CEA	4	Not reported separately	Not reported separately
Irqsusi et al. (2017)^[13]^	Cardiac surgery + CEA	4	Not reported separately	Not reported separately
Donatelli et al. (1998)^[34]^	Cardiac surgery + CEA	2	Not reported separately	Not reported separately
Perler et al. (1988)^[35]^	Cardiac surgery + CEA	1	Not reported separately	Not reported separately

## Conclusions

A wide range of severity and a wider spectrum of evidence exist regarding the management of coexisting carotid artery disease and cardiac pathology. Bilateral involvement, a higher degree of stenosis, and the presence of symptoms point toward the need for a carotid revascularization procedure. Low-quality evidence suggests that staged prophylactic CEA or carotid artery stenting followed by cardiac surgery yields favorable outcomes in terms of stroke and mortality, while a synchronous procedure decreases the risk for myocardial infarction.

There is a paucity of data regarding the management of concomitant severe aortic valve stenosis and carotid artery disease, and more data is required to frame suitable management guidelines. Transcatheter AVR might be a favorable alternative to surgical AVR for patients with a prior history of open heart surgery, although more data is required for a definitive recommendation. Nevertheless, carotid screening should be performed in patients with risk factors such as smoking, diabetes mellitus, PAD, and old age. In the absence of hard evidence, management plans should be individualized according to the patient's symptoms, the perioperative risk assessment, and the operating physician's competencies. More randomized controlled studies are required to establish guidelines for a definitive management strategy.
